# The *carB* Gene of *Escherichia coli* BL21(DE3) is Associated with Nematicidal Activity against the Root-Knot Nematode *Meloidogyne javanica*

**DOI:** 10.3390/pathogens10020222

**Published:** 2021-02-18

**Authors:** Yanfei Xia, Shen Li, Guohui Xu, Shanshan Xie, Xueting Liu, Xiaomin Lin, Huijun Wu, Xuewen Gao

**Affiliations:** 1 Department of Forensic Medicine, Affiliated Hospital of Guilin Medical University, 15 Lequn Road, Guilin 541001, Guangxi, China; ghxu2004@163.com; 2 Department of Plant Pathology, College of Plant Protection, Nanjing Agricultural University, 1 Weigang Road, Nanjing 210095, Jiangsu, China; xssflora871216@126.com (S.X.); gaoxw@njau.edu.cn (X.G.); 3 Department of Plant Protection, College of Horticulture and Plant Protection, Henan University of Science and Technology, 263 Kaiyuan Avenue, Luoyang 471023, Henan, China; 18438615966@163.com (S.L.); lytz1503678@163.com (X.L.); xiaomlin@163.com (X.L.)

**Keywords:** biocontrol, *Meloidogyne javanica*, *carB* gene, *Escherichia coli* BL21(DE3), culture filtrate

## Abstract

Biological nematicides have been widely used to lower the losses generated by phytoparasitic nematodes. The purpose of this study was to evaluate the nematicidal effects of *Escherichia coli* BL21(DE3) against *Meloidogyne javanica* and to identify nematicide-related genes. Culture filtrates of BL21(DE3) caused juvenile mortality and inhibited egg hatching in a dose-dependent manner. In the greenhouse, treatment of tomato seedlings with BL21(DE3) culture filtrates at 50 and 100% concentrations not only reduced the amount of *M. javanica* egg masses and galls, but improved plant root and shoot fresh weight. Culture filtrate analysis indicated that the nematicidal active ingredients of strain BL21(DE3) were non-proteinaceous, heat and cold resistant, sensitive to pH and volatile. To identify the genes associated with nematicidal activity, a BL21(DE3) library of 5000 mutants was produced using Tn5 transposase insertion. The culture filtrate of the MB12 mutant showed no nematicidal activity after 72 h of treatment and thermal asymmetrical interlaced PCR demonstrated that the *carB* gene was disrupted. Nematicidal activity was restored when the pH of the MB12 culture filtrate was adjusted to the original pH value (4.15) or following MB12 complementation with the *carB* gene, confirming a role for *carB* in mediating pH value and nematicidal activity. The outcomes of this pilot study indicate that BL21(DE3) is a potential microorganism for the continuable biological control of root-knot nematode in tomato and that *carB* affects the nematicidal activity of BL21(DE3) by modulating the pH environment.

## 1. Introduction

The root-knot nematode (RKN) *Meloidogyne* spp. is an important plant pathogen. It infects numerous crops, and leads to annual yield damages of about USD 400 million worldwide [1]. Chemical nematicides represent the conventional nematode management strategy due to their broad spectrum of targets and high efficacy [2]. However, some nematicides have been withdrawn or even banned, due to their latent poisonous effects on human and environmental safety [3]. Therefore, the search for novel potential candidates for the biocontrol of plant-parasitic nematodes to maintain economic and social stability has become increasingly important.

Numerous biocontrol bacteria (e.g., *Pasteuria* spp., *Bacillus* spp., *Pseudomonas* spp.,) have been proposed as sustainable alternatives to control phytoparasitic nematodes [4,5,6,7]. *Pasteuria penetrans* has been widely reported to directly parasitize plant-parasitic nematodes, especially *Meloidogyne* species [8,9]. Metabolic by-products from *Pseudomonas fluorescens*, such as 2,4-diacetylphloroglucinol (2,4-DAPG), have presented potential in killing cyst nematode juveniles [10,11]. *Bacillus nematocida* B16 possesses nematicidal activity against *Panagrellus redivius* as well as *Bursaphelenchus xylophilus*, using a Trojan horse mechanism [12,13]. Extracellular bacterial enzymes, which are critically important during nematode infection, rapidly decompose the cuticle of nematodes [14]. Moreover, *B. thuringiensis* (Bt) produces the well-known Cry protein, which displays specific toxic activity against nematodes and insects [15]. In addition, induced systemic resistance (ISR) is triggered by plant growth-promoting rhizobacteria (PGPR) [16,17]. 

*Escherichia coli* is the most widely studied prokaryotic model organism. The majority of *E. coli* strains are sackless, but a few serum types can result in critical illness in their hosts, such as enteropathegenic, enteroinvasive, enterohemorrhagic and enterotoxigenic *E. coli*. The *E. coli* strain OP50 is the famous nutrient material for *Caenorhabditis elegans* in the lab [18]. Recently, enterohemorrhagic *E. coli* O157:H7 was found to cause the death of *C. elegans* via either fast-kill or slow-kill phenotypes [19,20]. *M. javanica* acts as a provisional reservoir for *E. coli* strain TG1 under laboratory conditions [21]. The BL21(DE3) *E. coli* strain is widely used for the manufacture of recombinant proteins under the control of integrated T7-polymerase in biotechnology and microbiology [22]. However, to our knowledge, BL21(DE3) controlling plant-parasitic nematodes has not been described in the literature. We serendipitously observed that culture filtrates of BL21(DE3) showed strong nematicidal activity against RKNs and, to understand the nematicidal mechanism better, we studied this in more detail. Random DNA insertion into bacterial chromosomes is an important technique, which has been used to identify new genes, novel functions of known genes and to study protein functions. Recently, the Tn5-based transposon system has been successfully applied to mutagenize various bacteria to study genome evolution and genetic diseases [23,24,25]. The purpose of this research was to assess the nematicidal effects of *E. coli* strain BL21(DE3) culture filtrate against the RKN *M. javanica* and to identify potential nematicide-related genes using Tn5 transposon mutagenesis.

## 2. Results

### 2.1. In Vitro Nematicidal Activity

**Figure 1:**
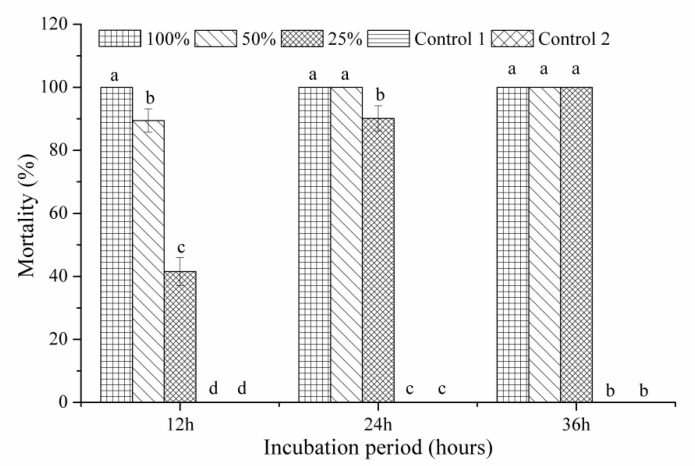
In vitro effect of different concentrations of *Escherichia coli* BL21(DE3) culture filtrates on *Meloidogyne javanica* mortality. Control 1: uninoculated Landy medium; Control 2: sterile distilled water. Data are means of one independent test with three replicates ± SE. Same letters demonstrate no statistical differences at *p* ≤ 0.05 by Fisher’s least significant difference test. Three independent tests produced similar outcomes; therefore, one result is shown.

**Figure 2:**
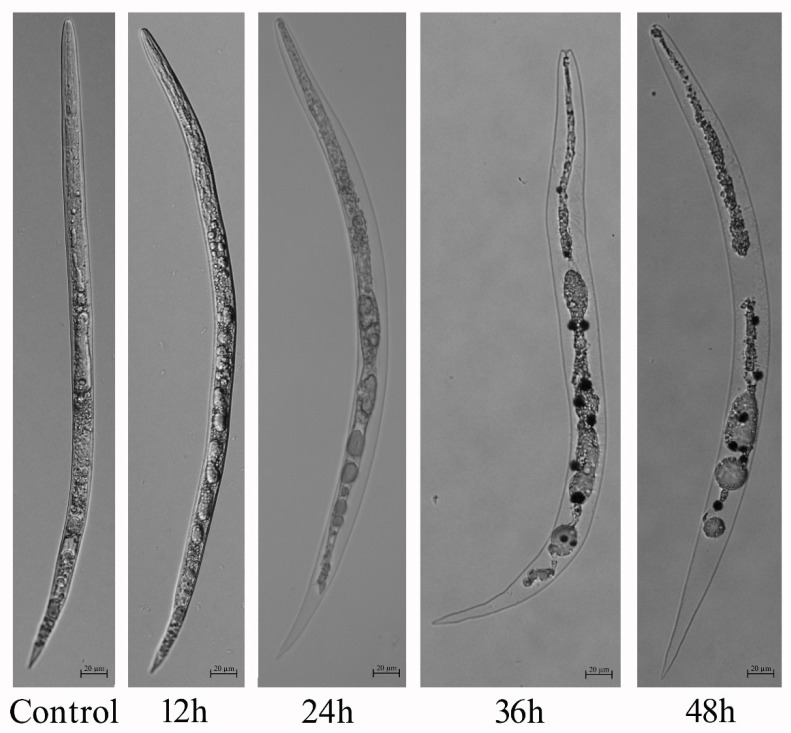
Morphological phenotypes of *Meloidogyne javanica* second-stage juveniles treated with undiluted supernatants (100% concentration) at different time points.

The percentage of nematode mortality differed with concentrations of culture filtrates of BL21(DE3) and exposure (Figure 1). The percentage mortality of second-stage juveniles (J2s) exposed to highly concentrated culture filtrates was markedly greater than that of those exposed to low-concentration culture filtrates and controls after 12 h of incubation. At this time point, 100% supernatants caused 100% J2 mortality. A 25% concentration of culture filtrate also led to 100% mortality of J2s following 36 h of incubation. Sterile distilled water and uninoculated Landy medium used as a control had no influence on the *M. javanica* juveniles examined. Generally, dead nematodes sank to the bottom, whereas those treated with BL21(DE3) culture filtrates were increasingly suspended in the culture filtrate as the treatment time increased. Furthermore, the internal organs of dead juveniles shrank and separated from the body cuticle and many bubbles were observed (Figure 2). No morphological changes were observed for J2s in the controls.

Disparate concentrations of BL21(DE3) supernatants remarkably suppressed the hatching of *M. javanica* eggs. However, J2 emergence was inversely proportional to the supernatant concentration. Exposure to increasing concentrations of BL21(DE3) culture filtrate increased the inhibition of egg mass hatching and inhibition rates ranged from 87.53% to 98.83% after two weeks of incubation (Table 1).

### 2.2. Nematicidal Active Stability

Following protease hydrolysis, the BL21(DE3) supernatants showed the same 100% nematicidal activity as the supernatants without protease treatment, suggesting that the nematicidal components are not proteins.

BL21(DE3) culture filtrates were subjected to cold or heat to examine the stability of the nematicidal metabolites. Despite these treatments, the extracts retained 100% nematicidal activity after an exposure period of 12 h, demonstrating that the active components are highly stable.

**Figure 3:**
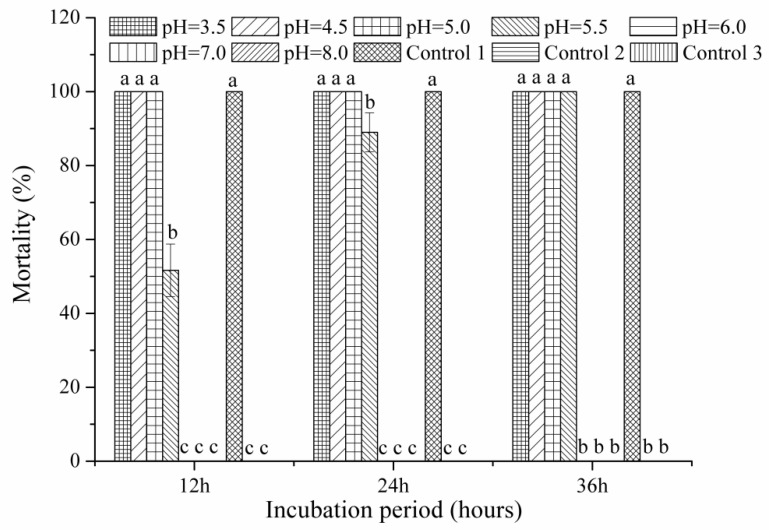
In vitro effect of pH-modified culture filtrates from BL21(DE3) on *Meloidogyne javanica* mortality. Control 1: original culture filtrate (4.15); Control 2: uninoculated Landy medium; Control 3: sterile distilled water. Data are means of one independent test with three replicates ± SE. Different letters indicate statistical differences at *p* ≤ 0.05 by Fisher’s least significant difference test. Three independent tests produced similar outcomes; therefore, one result is shown.

Following 12 h of incubation, BL21(DE3) culture filtrates with a broad pH range of 3.5–5.0 showed the same nematicidal efficacy as the original filtrates (Control 1), whereas those with a pH of 5.5, 6.0, 7.0 and 8.0 showed either reduced or no antagonistic activity (Figure 3). With an increase in exposure time, culture filtrates with a pH of 5.5 also resulted in 100% J2 mortality at 36 h, whereas those with a pH of 6.0, 7.0 and 8.0 still displayed no nematicidal activity. Sterile controls at pH 3.0 and 12.0 did not affect nematicidal activity. The outcomes indicate that the bioactive compounds in BL21(DE3) culture filtrates are stable in an acidic environment.

The collected extracts caused 100% mortality of J2s after 12 h of incubation, but the residues had no nematicidal activity after 48 h of incubation, suggesting that the nematicidal bioactive substances were volatile.

### 2.3. Glasshouse Experiments

**Figure 4:**
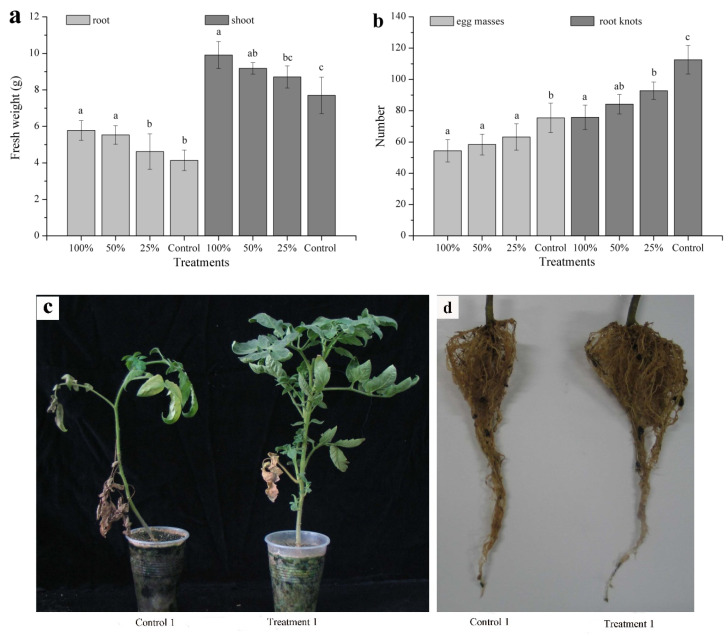
Effects of BL21(DE3) filtrate treatment on (**a**) tomato plant biomass, (**b**) *Meloidogyne javanica* root-knot and egg mass densities, (**c**) disease symptoms of shoot and (**d**) disease symptoms of root. Control: sterile distilled water. Control 1: tap water. Treatment 1: 100% concentration of BL21(DE3) culture filtrates. Values are the means ± SD from five replicates. For each parameter (root, shoot, egg masses and root knots), columns followed by the same letter are not significantly different at *p* ≤ 0.05 by Fisher’s least significant difference test.

Further studies were conducted to determine the effect of a soil drench of filtrates of strain BL21(DE3) against RKNs in tomato plants grown under greenhouse conditions. Following inoculation with 100% and 50% concentrations of BL21(DE3) culture filtrates, the root and shoot fresh weights increased by 39.61% and 28.70% (100% test), or by 33.57% and 19.22% (50% test), respectively (Figure 4a,c,d). The number of egg masses and root knots per plant decreased by 27.85% and 32.68% (100% test), or 22.55% and 25.22% (50% test), respectively (Figure 4b). Nevertheless, the 25% test only resulted in a significant difference in disease density and no remarkable difference in the biomass of tomato plants when compared to control plants (*p* ≤ 0.05).

### 2.4. Genetics of Nematicidal Activity

To identify and characterize potential genes associated with nematocidal activity, a transposon library containing 5000 BL21(DE) mutants was constructed by Tn5 random insertion and the library transformants were screened for nematicidal activity. One transformant, termed MB12, which produced a filtrate pH of 6.1, showed no nematicidal activity against *M. javanica* juveniles after 72 h of incubation.

The position of the Tn5 transposon in MB12 mutants was identified by thermal asymmetrical interlaced PCR. The PCR products were attached to a pUC18 vector and transformed into *E. coli* DH5α. The positive transformants were verified by PCR and sequenced. The insertion in MB12 was located in *carB*, which encodes a carbamoyl phosphate synthase (CPS) large subunit harboring two ATP-binding sites. The CPS enzyme catalyzes the conversion of bicarbonate, glutamine and two molecules of Mg^2+^ATP into carbamoyl phosphate (CP) [26].

The pColdcarB plasmid was constructed to confirm whether the *carB* gene was liable for the observed antagonistic effect of MB12. The MB12 mutant was transformed with pColdcarB and positive transformants were determined by PCR combined with a double digestion technique. The transformant named MB12-1, with a filtrate pH of 4.2, showed the same nematicidal efficacy as that foreseen in the wild-type strain BL21(DE3), demonstrating that the *carB* gene of *E. coli* BL21(DE3) mediated nematicidal activity. Furthermore, supernatants of MB12 adjusted to pH 4.15 also led to 100% mortality of J2s after 12 h of exposure.

## 3. Discussion

Many studies have reported that plant-parasitic nematodes can be controlled through active substances generated by bacteria during the fermenting process [7,11,13,17,27,28,29,30]. Specifically, several pathogenic *E. coli* isolates have been assessed for their ability to kill the free-living nematode *C. elegans* [19,20]. However, non-pathogenic *E. coli* that are antagonistic against *M. javanica* have not been documented. This study is the first to demonstrate nematicidal activity against the plant-parasitic nematode *M. javanica* by *E. coli* BL21(DE3) culture filtrates. Additionally, BL21 was also antagonistic to *M. incognita*, *M. arenaria*, *M. hapla* and *Heterodera glycines* in a similar way to that observed against *M. javanica,* but was not toxic to *Aphelenchoides besseyi*, *Ditylenchus destructor* and *Bursaphelenchus xylophilus* (data not shown). Similar results were reported by Mendoza et al. (2008), who demonstrated the effects of metabolic by-products of *Bacillus firmus* on *M. incognita*, *Radopholus similis* and *D. dispaci* [28]. The compound 2, 4-DAPG generated by *P. fluorescens* was not toxic to selected nematode [31]. At present, the mechanism for this disparity is not documented, but we speculate that the differences might reside in differences in the activity, detoxification, tolerance and exclusion capabilities between the different genera. Furthermore, supernatants of the virulent *E. coli* strain K88 and avirulent strains OP50, Top10, JM109, DH5α and Rosetta were also toxic to *M. javanica*, similar to the antagonistic effect on *M. javanica* presented in this research, and all these strains showed a similar nematicidal activity (data not shown). The findings suggest that the nematicidal active components are present in supernatants of these isolates. Indeed, some bioactive substances, such as lytic enzymes, Cry toxins, 2-hydroxypropanoic acid, plantazolicin, 2, 4-DAPG and sphingosine isolated from bacterial culture filtrates, are well known to antagonize plant-parasitic nematodes [11,15,32,33,34]. Analysis of BL21(DE3) culture filtrates indicated that the bioactive compounds were non-proteinaceous, heat and cold resistant and volatile, similar to the active components produced by *B*. *subtilis* strain OKB105 [29]. The data here clearly indicate that pH affected the antagonistic nematicidal activity of these compounds. Similarly, sphingosine produced by *B. cereus* strain S2 displayed nematicidal potential in the pH range from 2.0 to 8.0 [33]. Additionally, BL21(DE3) nematicidal compounds destroyed the internal structure of J2s and numerous large vesicles were observed in J2s treated with BL21(DE3) supernatants. Similar symptoms of internal nematode organ injury have been documented following treatment with *B. cereus* X5, *Camellia* seedcake extracts, 5-Aminolevulinic acid (ALA) or sphingosine [30,33,35,36]. Further research is required to identify the nematicidal substances, using analytical techniques such as liquid chromatography–mass spectrometry (LC-MS), gas chromatography–mass spectrometry (GC-MS) and nuclear magnetic resonance (NMR), and to elucidate the nematicidal mechanism of BL21(DE3).

On account of the growth-promoting capability and the nematicidal potential, numerous bacteria (e.g., *Bacillus* spp., *Pseudomonas* spp.) can be used to manage plant-parasitic nematodes via various mechanisms of action [7,37]. However, to our knowledge, no reports exist concerning the use of BL21(DE3) as a biocontrol agent. The results obtained under greenhouse conditions demonstrated that BL21(DE3) filtrates increased the growth parameters of tomato seedlings (root and shoot fresh weight) and decreased the harm degree generated by *M. javanica* (amount of egg masses and root knots) when used at a concentration of 50 or 100%, whereas treatment with a 25% BL21(DE3) filtrate concentration was ineffective on tomato growth compared to the control. These data therefore indicate that a 50% BL21(DE3) filtrate concentration is optimal to control *M. javanica* in a greenhouse environment; nevertheless, further research is necessary to test the biocontrol effect of BL21(DE3) against RKNs in field trials and to demonstrate the mechanism of action against nematodes in vivo.

Random mutagenesis systems based on in vivo transposon insertion into the genome are extensively applied to mining functional genes of microorganisms [38,39]. In this research, the BL21(DE3) mutant library was obtained using the Tn5 transposon and the mutant nematicidal capability was assessed to identify nematicide-related genes. The MB12 mutant showed a lack of nematicidal activity and 14 mutants delayed the onset of injury to the internal organs of dead J2 nematodes (data not shown). The Tn5 transposon of MB12 was located within the *carB* gene, which encodes a large subunit of CPS. Carbamoyl phosphate is a vital small molecule that powers cell metabolism and can donate a carbamyl group within numerous well-known biosynthetic pathways, including the arginine/urea, pyrimidine and antibiotic synthesis pathways [40,41]. Besides being involved in biosynthetic pathways, *carB* has been recently reported to possess novel functions: disruption of *carB* in *Xanthomonas citri* subsp. *citri* caused pathogenic loss and an inability to induce a hypersensitive reaction (HR) in non-host plants, but also led to reduced swimming ability and biofilm formation [42,43]. The *carB* gene of *Toxoplasma gondii*, which results in birth defects and destructive toxoplasmic cephalitis in immunocompromised humans, has an important role in growth and virulence [44]. The findings are the first to demonstrate that the *carB* gene of BL21(DE3) affects nematicidal capability. The *cheB2*, *mucD* and *purL* genes are also involved in nematicidal activity [29,45,46]. Loss of *carB* function in the MB12 mutant affected the synthesis of CPS, the amino acid sequence of which differs from those nematicidal proteases of bacteria [14,47,48]. This finding, in combination with the properties of supernatants obtained in this study, demonstrates that the large CPS subunit was not responsible for nematicidal activity. Culture filtrates of the complemented MB12-1 strain displayed the same nematicidal capability as the original BL21(DE3) supernatants, but also a similar pH value. The nematicidal activity of MB12 was restored following adjustment of the pH to the control pH value of 4.15. These results, in association with the pH stability of supernatants demonstrated here, indicate that BL21(DE3) nematicidal activity is indirectly affected by *carB* via a change in pH. A similar study has reported that *carB* of *X. citri* subsp. *citri* affected pathogenicity and HR in non-hosts by regulating *hrp* gene expression [42]. Furthermore, the *purL* gene had been shown to mediate the antagonistic potential of *B. subtilus* strain OKB105 via the metabolic intermediates of purine biosynthesis [29]. The data here indicate that *carB* regulates the nematicidal ability of BL21(DE3) by modulating the pH environment. However, the mechanism remains indistinct and requires further study.

In conclusion, this study is the first report that *E. coli* strain BL21(DE3) is a potential microbe for the management of RKNs in tomato. We also demonstrated that the loss of *carB* function led to the loss of nematicidal activity of BL21(DE3) by altering the pH environment.

## 4. Materials and Methods

### 4.1. Strains and Plasmids

The bacterial strains and plasmids used for this study are listed in Table 2. *E. coli* strain DH5α acted as the host for all plasmids. Luria broth (LB) was used for the growth of all *E. coli* strains and BL21(DE3) mutants at 37 °C. Landy medium was used to ferment *E. coli* BL21(DE3) and mutants [49]. All of the strains were stored at −80 °C. A small piece of ice carrying the strain was taken from the stores by using a sterilized toothpick, streaked on LB medium and then cultured at 37 °C. After 24 h of incubation, the single colony, whose phenotype showed no difference from the previous strain, was transferred to liquid medium for further research. Where necessary, media were supplemented with the corresponding antibiotics (ampicillin at 100 ug mL^−1^ and kanamycin at 50 µg mL^−1^).

### 4.2. Preparation of Meloidogyne javanica

*M. javanica* was originally obtained from Hainan province, China and has been cultivated continually in tomato plants. The egg masses were gathered from roots of glasshouse-grown tomato plants and the second-stage juveniles (J2s) were separated by the Baermann funnel technique [50]. The collected eggs and nematodes were separately sterilized using 1% NaClO for five minutes and washed three times with sterile distilled water before use.

### 4.3. Antagonism of Strain BL21(DE3) against M. javanica In Vivo

The BL21(DE3) strain and mutants were grown in Landy medium on a rotary shaker (200 rpm) at 37 °C for 48 h. Culture filtrates were collected at 12,000 rpm for 15 min at 4 °C and disinfected using 0.22 μm bacterial filters; these were denoted as original supernatants (100% concentration) that were successively attenuated with sterile distilled water to 50 and 25% concentrations to observe nematicidal activity following the described procedure [29]. The number of dead nematodes was recorded using a light microscope at 12, 24, 36 and 48 h. Uninoculated Landy medium and sterile distilled water were used as controls. Tests were performed three times with three replicates per trial.

To assess the ovicidal action of BL21(DE3), egg masses were prepared following a revised method [51]. Briefly, six disinfected egg masses of the same size were stochastically picked using sterile forceps and placed into a 6 cm diameter watch glass with 1 mL 100%, 50% or 25% concentrations of BL21(DE3) supernatant. Sterile distilled water was used as a control. All treatments were placed in a 25 °C incubator for two weeks. The incubation solutions were changed for fresh ones every two days. The solution containing the nematodes was transferred to a counting dish and the J2 number was recorded under a dissecting microscope. Tests were performed three times with three replicates per treatment.

### 4.4. Different Treatments of BL21(DE3) Supernatants

The BL21(DE3) filtrates were treated with trypsin or proteinase K (20 mg mL^−1^) at 37 °C for thirty minutes. Thereafter, the nematicidal capability of the supernatants was observed according to the method above. The cold and heat resistance of the bacterial metabolites was based on previously described procedures [27,29]. Culture filtrates were separately incubated at 4 °C, −25 °C and −80 °C for 10, 20, 30, 60 or 90 days; at 100 °C for 10, 20 or 30 min; or at 121 °C for 10, 15 or 20 min in an autoclave and were successively examined for nematicidal capability according to the method above. For testing pH stability of BL21(DE3) filtrates, supernatants were calibrated at pH 3.5, 4.5, 5.0, 5.5, 6.0, 7.0 or 8.0 and were examined for nematicidal activity. The original supernatants (pH 4.15) and sterile distilled water with pH 3.0 and 12.0 were used as controls. To assess the volatility of the nematicidal principles, 100 mL supernatants were evaporated to dryness at 60 °C using a rotary evaporator. Obtained distillations and residues from the initial filtrates were diluted to the original volume (100 mL) and were evaluated for nematicidal capability.

All the above experiments were repeated three times with three replicates per treatment.

### 4.5. Antagonism of Strain BL21(DE3) against M. javanica In Vivo

Since strain BL21(DE3) led to a high fatality rate of J2s and inhibited the hatching of egg masses, it was selected to further evaluate its biocontrol potential in a pot test. Tomato seeds (*Solanum lycopersicum* cv. Sufen) were disinfected with 1% NaClO for 5 min and washed a few times with distilled water. The soil used in these trials consisted of a 1:1 (*v/v*) mixture of sterilized sand and silt loam. Treated seeds were grown in a tray and, 7 days after sprouting, an individual seedling was cultivated in a 10 cm diameter plastic pot. At 40 days after transplanting, 25 mL supernatants were poured onto the root system of each plant using a soil drench. Tap water served as a control treatment. After 24 h, 1000 newly hatched J2s per tomato were injected into a 3 cm deep hole. The test was performed thrice, and each treatment consisted of five replicates, which were randomly put in a glasshouse. These tomatoes needed watering daily and manuring weekly. The tests ended 50 days after inoculation and the fresh weight of the roots and shoots was measured. The number of egg masses and galls per plant was also recorded.

### 4.6. DNA Manipulation and Transformation

DNA ligases and restriction enzymes were applied according to the manufacturer’s instructions (TaKaRa, Japan). Kits for isolating and purifying DNA were purchased from Axygen Scientific Inc. (USA). *E. coli* strain DH5α was transformed as described [52]. The specific primers are listed in Table 3.

The BL21(DE3) mutant library was prepared using the transposase–transposon complex (EZ-Tn5^TM^<R6Kγori/KAN-2>Tnp Transposome^TM^ kit) according to the manufacturer’s instructions (Epicentre, Madison, WI, USA). Transformation of cells with DNA was performed by electroporation (1800 V, 24 μS, 200 Ω, 0.1-cm cuvettes). The cells were renatured by incubation at 25 °C for 5 min with 200 µL LB liquid medium, then put in a 1.5 mL centrifuge tube with shaking (200 rpm) at 37 °C for 1.5 h. The transformants were plated on LB plates with 50 μg mL^−1^ kanamycin and cultured at 37 °C for 12 h. Every transformed colony was individually recorded and transferred onto new LB plates with kanamycin for further study. To create a transposon library, 10 individual electrotransformations were carried out as described above. The correspondingly numbered clones were stored in 2.0 mL centrifuge tubes with 30% glycerol at −80 °C. The nematicidal activity of mutants was evaluated according to the method above.

### 4.7. Determination of the Transposon Insertion Site

A fragment flanking the Tn5 transposon was amplified with transposon-specific primers and six arbitrary degenerate primers (Table 3) by thermal asymmetrical interlaced PCR (TAIL-PCR). The reactions were carried out with a T100^TM^ Thermal Cycler (Bio-Rad, USA) following the described procedure [53]. Amplified fragments were purified and sequenced. The nucleotide sequences were aligned with the complete genome sequences of *E. coli* strain BL21(DE3) via NCBI BLAST (http://www.ncbi.nlm.nih.gov/BLAST (accessed on 20 January 2021)) to confirm the Tn5 insertion site in the mutant.

### 4.8. Construction of Complementation Plasmid

For MB12 mutant complementation, the pColdcarB plasmid was obtained as follows. The complete sequence of the *carB* gene was amplified from strain BL21(DE3) chromosomal DNA using primers carB-F/carB-R. After purification, the DNA fragment was cloned into the pUC18 vector to create the pUCcarB plasmid. After the integrity of the open reading frame was confirmed by sequencing, the full-length *carB* gene was obtained using restriction endonucleases *Bam*HI and *Hin*dIII, purified and attached to pColdII to create the expression vector pColdcarB. The plasmid pColdcarB was shifted to MB12 and picked on LB agar with 100 µg mL^−1^ ampicillin and 50 µg mL^−1^ kanamycin. *M12::pColdcarB* was identified by PCR with primers pColdII-F/pColdII-R and *Bam*HI/*Hin*dIII double digestion, and the correct transformant was named MB12-1. The *cspA* promoter was used to express *carB* in MB12-1.

### 4.9. Statistical Analysis

Data were assessed by analysis of variance using SPSS software (SPSS Inc., Chicago, IL, USA). Different letters in the figures indicate significant differences with Fisher^’^s least significant difference test (*p* ≤ 0.05) between treatment and control.

## Figures and Tables

**Table 1:** Effect of different concentrations of *Escherichia coli* BL21(DE3) on the cumulative hatching frequency of egg masses of *Meloidogyne javanica* after 14 days of incubation in vitro.

Treatment	No. of Juveniles Hatched	Hatching Inhibition Rate (%)
100%	28.67 ± 7.37 a	98.83
50%	133.33 ± 18.61 b	94.56
25%	305.67 ± 34.39 c	87.53
Control	2453 ± 121.20 d	

Control, sterile distilled water. Values are means ± SD. In a column, data followed by different superscript letters are significantly different at *p* ≤ 0.05 by Fisher’s least significant difference test. Three replicates were performed per treatment. Three independent tests produced similar outcomes; therefore, one result is shown.

**Table 2:** Bacterial strains and plasmids used in this study.

Material	Relevant Genotype or Characteristics *	Source or Reference
**Strains**		
*Escherichia coli*		
DH5α	*F^-^, φ80d/lacZΔM15*, *Δ*(*lacZYA-argF*)U169,*deoR*	This lab
BL21(DE3)	*F^-^, ompT, hsdSB*(*rB- mB-), gal dcm*(DE3)	This lab
MB12	Mutant of BL21(DE3), *carB*::Tn5; Km^r^	This study
MB12-1	Complementation of MB12 mutant with expression vector pCold*carB;* Ap^r^, Km^r^	This study
**Plasmids**		
pUC18	*E. coli* clone vector; *lacZ*; Ap^r^	This lab
pColdII	*csp*A promoter-based expression vector; Ap^r^	This lab
pUCcarB	pUC18 derivative carrying *carB*; Ap^r^	This study
pColdcarB	pCold II derivative carrying *carB*; *cspA* promoter-based expression vector; Ap^r^	This study

* Resistance marker: Ap^r^, ampicillin resistance; Km^r^, kanamycin resistance.

**Table 3:** Oligonucleotide DNA primers used in this study.

Primers	Sequence (5′-3′; Restriction Sites Underlined if Present)	Use of Primers
carB-F	GGATCCATGCCAAAACGTACAGATAT (*Bam*HI)	*carB* gene amplification
carB-R	AAGCTTTTATTTGATCTGTGCGTGCA (*Hin*dIII)	
pColdII-F	ACGCCATATCGCCGAAAGG	*carB* gene identification
pColdII-R	GGCAGGGATCTTAGATTCTG	
SP1	GTCTTCGGTTTCCGTGTTTCG	Tn5 transposon left flanking sequences Pre-amplification
DP1	NGTCGASWGANAWGAA	
SP2	AAATGGCATCCGGATCTGCATC	Tn5 transposon left flanking sequences Primary amplification
DP2	AGWGNAGWANCAWAGG	
SP3	TACCCTGTGGAACACCTACATCTG	Tn5 transposon left flanking sequences Second amplification
DP3	CAWCGICNGAIASGAA	
SP4	GGTTGTAACACTGGCAGAGCATT	Tn5 transposon right flanking sequences Pre-amplification
DP4	TCSTICGNACITWGGA	
SP5	CGCATCTTCCCGACAACGCAG	Tn5 transposon right flanking sequences Primary amplification
DP5	STTGNTASTNCTNTGC	
SP6	AACTGGTCCACCTACAACAAAG	Tn5 transposon right flanking sequences Second amplification
DP6	WCAGNTGWTNGTNCTG	

Nested sequence-specific primers (SPs) SP1, SP2 and SP3 are located at the left border of the Tn5 transposon; SP4, SP5 and SP6 are at the right border of the Tn5 transposon. DP1, DP2, DP3, DP4, DP5 and DP6 are shorter arbitrary degenerate primers (DPs).

## Data Availability

Data sharing not applicable.
